# Towards personalized induction therapy for esophageal adenocarcinoma: organoids derived from endoscopic biopsy recapitulate the pre-treatment tumor

**DOI:** 10.1038/s41598-020-71589-4

**Published:** 2020-09-03

**Authors:** Mathieu F. Derouet, Jonathan Allen, Gavin W. Wilson, Christine Ng, Nikolina Radulovich, Sangeetha Kalimuthu, Ming-Sound Tsao, Gail E. Darling, Jonathan C. Yeung

**Affiliations:** 1 Latner Thoracic Surgery Research Laboratories, Princess Margaret Cancer Research Tower, University Health Network, Toronto, ON Canada; 2 Princess Margaret Cancer Centre, University Health Network, Toronto, Canada; 3 Department of Pathology, University Health Network, Toronto, Canada; 4 Division of Thoracic Surgery, Toronto General Hospital, University Health Network, 200 Elizabeth St, 9N-983, Toronto, ON M5G 2C4 Canada

**Keywords:** Oesophageal cancer, Cancer models

## Abstract

Esophageal adenocarcinoma has few known recurrent mutations and therefore robust, reliable and reproducible patient-specific models are needed for personalized treatment. Patient-derived organoid culture is a strategy that may allow for the personalized study of esophageal adenocarcinoma and the development of personalized induction therapy. We therefore developed a protocol to establish EAC organoids from endoscopic biopsies of naïve esophageal adenocarcinomas. Histologic characterization and molecular characterization of organoids by whole exome sequencing demonstrated recapitulation of the tumors’ histology and genomic (~ 60% SNV overlap) characteristics. Drug testing using clinically appropriate chemotherapeutics and targeted therapeutics showed an overlap between the patient’s tumor response and the corresponding organoids’ response. Furthermore, we identified Barrett’s esophagus epithelium as a potential source of organoid culture contamination. In conclusion, organoids can be robustly cultured from endoscopic biopsies of esophageal adenocarcinoma and recapitulate the originating tumor. This model demonstrates promise as a tool to better personalize therapy for esophageal adenocarcinoma patients.

## Introduction

Esophageal adenocarcinoma (EAC) remains one of the least studied and most lethal malignancies with an overall 5 year survival rate of only around 18%. The high case-fatality ratio of EAC is a consequence of the limited treatment options available and the limited avenues to personalize treatment. Multiple treatment regimes (e.g. CROSS, FLOT) currently exist, but which tumor best responds to which regimen remains unpredictable and current selection protocols focus on location of tumor and patient performance status rather than any biological factor^1–4^. Ideally, individual patients’ tumors would be tested against the different potential induction strategies prior to starting treatment to identify the most effective treatment regimen; however, the ex vivo culture of patient tumors has been limited. Establishment of EAC cell lines from patient tumors is time consuming, often unsuccessful, and the immortalization process likely alters the molecular characteristics of the tumor^5, 6^. Patient-derived xenografts have been much more successful in other cancers, but the success rate with EAC has been quite low at < 30% and the culture time makes it unfeasible for testing pre-surgical therapy^7^. Overall, the culture of EAC tissue ex vivo for therapeutic testing has been challenging.

The development of organoid technology demonstrates promise to fill this gap in EAC research. In other disease sites, patient-derived organoids have been cultured in a clinically relevant timeframe, shown to recapitulate the primary tumor at a molecular level, and respond to certain chemotherapeutics similar to the tumor^8, 9^. Recently, Li et al. demonstrated the successful generation of EAC organoids that recapitulate the original tumor and used them to test novel therapeutic approaches^10^. However, these EAC organoid models were generated from surgical specimens at the time of cancer resection and therefore represent residual tumors following an induction regimen, rather than the original tumor. As surgery represents the best chance for disease clearance, we feel that a more valuable approach for using EAC organoids would be to identify the induction therapy that will best shrink the tumor prior to surgery. To that end, we believe the best time to culture EAC organoids is at the time of diagnosis so that induction treatment options can be tested in organoids prior to use in the patient. In this study, we aimed to test this concept in EAC by assessing the effectiveness of organoid culture from endoscopic biopsies taken at the time of diagnosis, assessing the molecular overlap between the resulting organoids and primary tissue, and evaluating the relative response of endoscopic biopsy derived organoids (EDO) to chemotherapeutic agents when compared to the originating patient’s response.

## Results

From July 2017 to July 2018, using our organoid culture protocol, we successfully grew 16 EDO from 28 processed tissues for an establishment success rate of 57.2%. The main issues limiting the success rate were bacterial contamination (21.4%) and non-growth (21.4%). To assess whether EDO can recapitulate the heterogeneity of EAC tumors, we picked 5 tumors with distinctive morphological features and treatment heterogeneity (Tables 1 and 3). Those selected were then processed for full characterization and drug testing. Our first level of validation consisted of short tandem repeat (STR) analysis, histology, and immunohistochemistry. These assays were performed after the 6th passage. The STR overlap ranged from 78 to 94% (Table 2). The mean doubling time was around 94 h (ranging from 56 to 150 h) (Table 2).

**Table 1:** Patient clinical characteristics.

Study number	Year of birth	Gender	cT	cN	cM	Bx diagnosis	HER-2	Grade	Treatment response/effect	Treatment regimen	Organoid initiation
46^b^	1963	Male	T2	N1	M0	Adenocarcinoma	Negative	G2	Grade 2	CROSS	Success
50^a^	1960	Male	T2	N0	M0	Adenocarcinoma	Not done	G2	Grade 2	CROSS	Success
51	1949	Male	T3	N0	M1	Adenocarcinoma	Negative	n/a	n/a	n/a	Fail (no growth)
52^a^	1954	Male	T3	N0	M0	Adenocarcinoma	Negative	Not described	Grade 3	CROSS	Success
53	1955	Male	T3	N0	M0	Adenosquamous carcinoma	Negative	Not described	Grade 3	FLOT	Fail (contamination)
54	1953	Female	T3	N1	M0	Adenocarcinoma, invasive	Positive	G2	Grade 2	CROSS	Success
55^a^	1973	Male	T2	N0	M1	Adenocarcinoma	Negative	GX	Grade 1	CROSS	Success
57	1964	Male	T3	N1	M0	Adenocarcinoma	Negative	G2	Grade 2	CROSS	Success
58	1978	Male	T3	N2	M0	Adenocarcinoma	Negative	n/a	Grade 3	FLOT	Fail (contamination)
61	1940	Male	T3	N1	M1	Adenocarcinoma	Negative	n/a	n/a	CROSS	Fail (contamination)
62	1973	Male	T3	N1	M0	Adenocarcinoma	Positive	G2	Grade 2	CROSS	Fail (no growth)
64	1960	Male	T3	N1	M0	Adenocarcinoma	Negative	G2	Grade 1	CROSS	Success
68	1943	Male	T3	N0	M0	Adenocarcinoma	Negative	Not described	Grade 2	FLOT	Success
70^b^	1954	Male	T3	N2	M0	Adenocarcinoma, invasive	Negative	G3	Grade 2	FLOT	Success
71	1957	Male	T3	N1	M0	Adenocarcinoma	Negative	G3	Grade 3	FLOT	Fail (contamination)
73	1946	Male	T3	N0	M0	Adenocarcinoma	Negative	G2	Grade 2	ECF	Fail (no growth)
74^b^	1957	Male	T2	N0	M0	Adenocarcinoma	Positive	G2	n/a	Surgery only	Success
76	1952	Male				GIST tumour (has liver mets)	n/a	n/a	n/a	GLEEVAC	Success
77^b^	1939	Male	T3	N0	M0	Adenocarcinoma	Positive	G2	n/a	Surgery only	Success
78^b^	1961	Male	T3	N2	M0	Adenocarcinoma	Negative	G3	Grade 2	CROSS	Success
79^b^	1988	Male	T3	N1	M0	Adenocarcinoma	Negative	G3	Grade 3	FLOT	Success
82^b^	1963	Male	T3	N1	M0	Adenocarcinoma	Negative	G2	Grade 1	FLOT	Success
83	1950	Male	T3	N1	M0	Carcinoma with occasional signet ring cells	Not tested	G3	Grade 1	FLOT	Fail (contamination)
84	1950	Male	T3	N1	M0	Adenocarcinoma, Barrett's	Positive	GX	Grade 1		Fail (no growth)
85	1937	Male	T3	N2	M1	Adenocarcinoma	Negative	n/a	n/a	Palliative radiation	Fail (no growth)
86	1949	Male	T3	N1	M1	Adenocarcinoma, invasive	Negative	n/a	n/a	Palliative radiation	Fail (no growth)
89	1953	Male	n/a	n/a	n/a	No tumour, only peptic stricture	n/a	n/a	n/a	No treatment	Fail (contamination)
92^b^	1951	Male	T3	N0	M0	Adenocarcinoma, gastric polyposis	Negative	G3	Grade 1	CROSS	Success

G2: Moderately Differentiated, G3: Poorly differentiated, undifferentiated, Gx: could not be assessed, Grade 1: Near complete response, Grade 2: Partial response, Grade 3: no response, CROSS: Carboplatin and Paclitaxel, FLOT: 5FU, Leucovorin, Oxaliplatin and Docetaxel.

^a^Denotes organoid culture which have been validated via immunohistochemistry.

^b^Denotes organoid culture which have been validated with STR, immunohistochemistry and exome sequencing.

**Table 2:** Summary of the characteristics of the endoscopic biopsy derived organoids.

EDO	STR (%)	Doubling rate (h)	Medium	p53 staining (organoid)	p53 staining (endo)	CK7 staining (organoid)	CK7 staining (endo)	Split ratio	Passage interval (days)
46	87	150	A	Positive	Positive	Positive	Positive	1:6	7
70	78	56	A	Focal	Focal	Positive	Positive	1:3	6
74	97	120	B	Positive	Positive	Positive	Positive	1:4	8
82	86	73	B	Positive	Positive	Negative	Negative	1:2	8
92	94	69	A	Positive	Positive	Negative	Positive	1:4	10

The primary tumour in the 5 endoscopic biopsies showed a spectrum of morphological features ranging from well formed glands to cribriform nests to sheets of cells, demonstrating the presence of morphological heterogeneity. Overall, the EDOs recapitulated the cytological features of the endoscopic biopsies; however, architecturally, all organoids demonstrated minimal variation but did differ in the size and number and size of lumina (Table 3 and Supplementary Fig. S1).

**Table 3:** Summary table of morphological features of endoscopy biopsies and matched EDO.

Patient	Morphology-endoscopic biopsy	Tumour cellularity in endoscopic biopsy (%)	Morphology-organoid
46	Invasive adenocarcinoma comprising large, cribriform nests of cells with "comedo type" necrosis and high N:C ratio	80	Clusters of cells (ranging from 5 to 40 cells) show lumina of varying sizes, including some cystic dilatation, cytomorphologically similar to the primary tumour. Occasional clusters show the presence of multiple lumina, recapitulating the cribriform architecture of the primary tumour
70	Squamous esophageal mucosa with moderately differentiated carcinoma, comprising infiltrating tubular glands and cords, seen undermining the overlying epithelium. The cells have high N:C ratio and moderate amounts of eosinophilic cytoplasm	20	Small clusters of cells (up to 10–15 cells) containing a central lumina, cytomorphologically similar the primary tumour
74	Invasive moderately differentiated adenocarcinoma with a tubulopapillary architecture and high grade cytology, on a background of Barrett's esophagus	60	Clusters of cells (ranging from 5 to 60 cells) with central lumina with cytomorphology similar to the primary tumour and associated necrotic debris. Occasional coalescent clusters are seen
82	Columnar lined epithelium with underlying infiltrating, invasive poorly differentiated carcinoma comprising predominantly of sheets of cells with high N:C ratio, angulated nuclei, focal intracytoplasmic vacuolation and only focally forming abortive lumina	10	Clusters of cells (up to 50–60 cells), some of which contain lumina of varying sizes with cytomorphological features similar to the primary tumour. In addition, focal evidence of apoptotic debris/necrosis is also seen
92	Small polypoid fragment of squamous mucosa with underlying poorly differentiated carcinoma, with single cells, some of which, demonstrating a signet ring cell morphology	30	Clusters of cells (ranging from 10 to 100 cells) show tightly packed cells, which are cytomorphologically similar to the primary tumour and occasional cells are also seen to contain intracytoplasmic mucin

**Figure 1:**
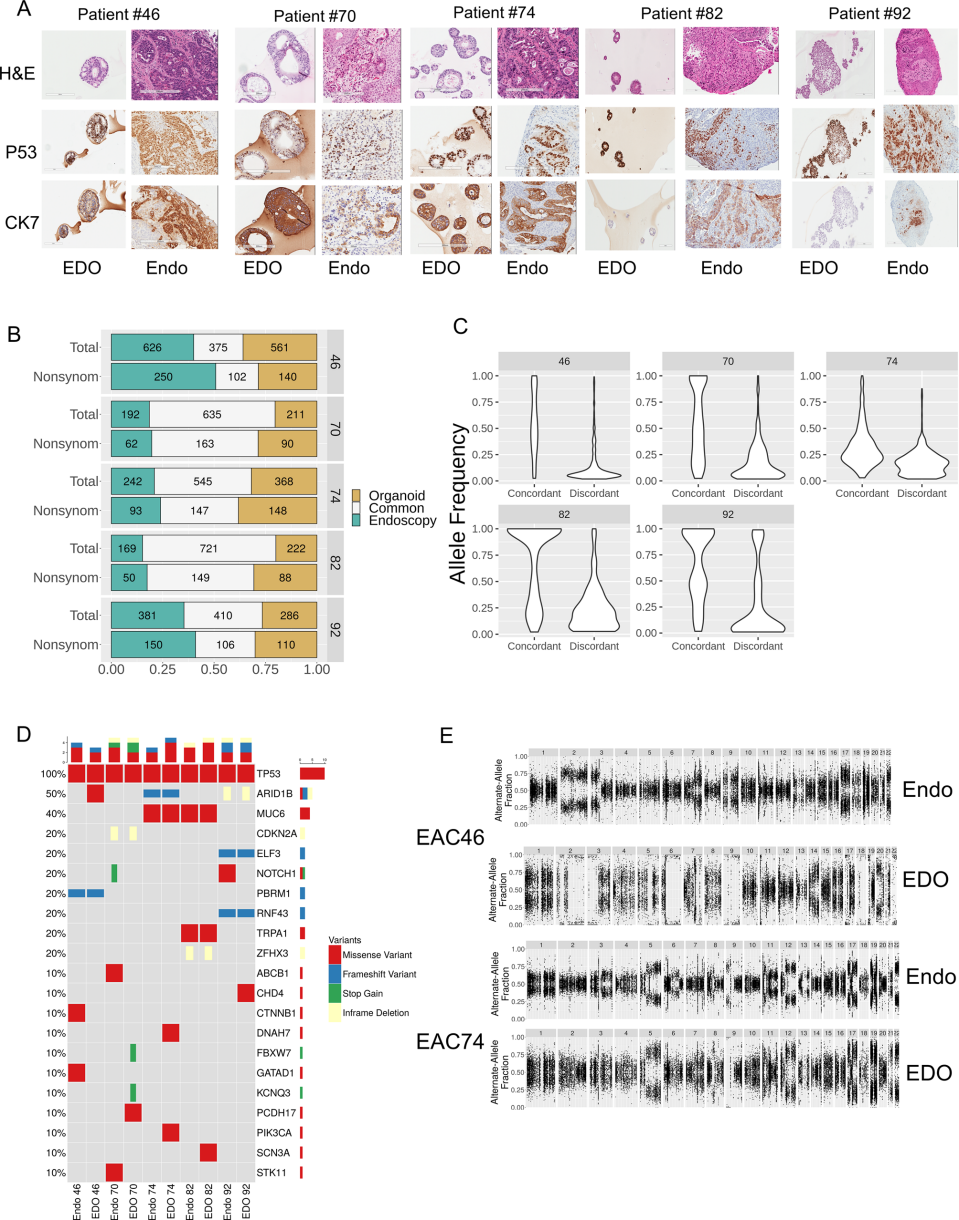
Histological and genomic characterization of the EDOs. (**A**) Representative images of H&E and IHC of P53 and CK7 between all 5 pairs of endoscopic biopsy and organoid. (**B**) Concordance and discordance plots of SNVs for both endoscopy tumors and EDO. (**C**) Violin plots of the frequency plots of concordant and discordant SNVs for both endoscopy tumors and EDOs. (**D**) Oncoprint table for all 5 paired samples. The gene list was established from previous publication (see “Methods”). (**E**) Copy Number Variation plots of patient 46 and 74 paired samples (endoscopy and EDO).

On immunohistochemistry, all five organoids demonstrated similar p53 staining as the original tumor. Concordance between the tumor and EDO was found in patients 46, 70, and 74 (Fig. 1A and Table 2). However, in patient 92, the organoid was negative for Cytokeratin 7 (CK7) staining while the tumors had some positive staining. This may be a result of tumor heterogeneity or tumor evolution. Overall, the EDO appear to recapitulate most histological features of their respected tumors.

### Genomic comparison of the tumor and EDO

We performed whole exome sequencing (WES) on our five patients. For each patient, we processed DNA from blood, the tumor, and EDO (past passage 6). The average concordance of short indels and SNVs between EDO and paired endoscopic biopsy was increased from a mean of 30.1% to 64.1% by “rescuing” the low confidence calls of SNV from the tumor exomes when comparing to the organoid high confidence calls of SNVs, suggesting tumor heterogeneity (Fig. 1B). The SNV allele frequency of the unique mutations was largely between 0.05 and 0.25, whereas the frequency of the shared mutations was spread from 0 to 1 (Fig. 1C), again suggesting heterogeneity.

To investigate which genes were commonly mutated between the tumors and organoids, we used a previously published EAC driver genes list^11^. TP53 was mutated in all 5 sets of samples, even in patient 70 which displayed a wild-type expression pattern on IHC. Each tumor-organoid pair had a different TP53 mutation (Supplementary Fig. S1). Outside of TP53, other mutations shared with the EAC driver gene list included MUC6 (EDO 74 and 82), CDKN2A (EDO 70) and ARID1B (EDO74 and 92) (Fig. 1D). When we looked at all SNV, we discovered previously undescribed mutations shared between tumor-organoid pairs, such as AHNAK2 and LAMA1 (shared between EDO 74 and 82) and LILBR2 (shared between EDO 92 and 74) (Supplementary Fig. S2).

Lastly, we compared copy number variations (CNV) between tumor-organoid pairs. In two of our endoscopic tumor samples (EDO 70 and EDO 82), normal tissue contamination complicated comparisons and this was reflected by the low tumor cellularity of the adjacent endoscopic biopsy (Table 3, Supplementary Fig. S2). EDO 46 and EDO 74 demonstrated good overlap (Fig. 1E). In EDO 92, and to an extent 82, the EDO demonstrated a significant loss of heterozygosity (Supplementary Fig. S2). This could be an indication of a sub-population of cells present in the tumor which outgrew the other cancer cells and correlates with the disparate CK7 seen on IHC.

### Drug treatment response of organoids reveals similarity with tumor response

**Figure 2:**
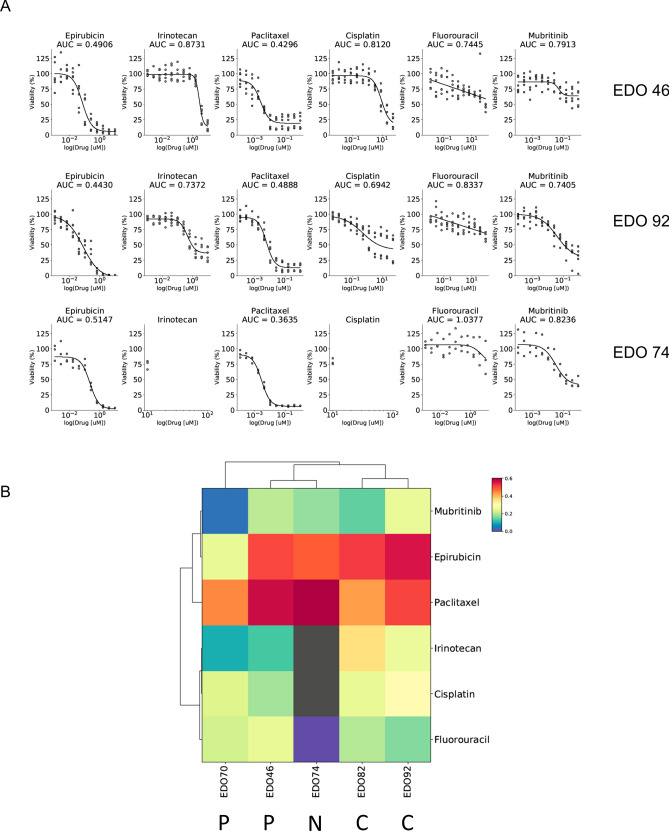
EDO sensitivity to chemotherapy drugs. (**A**) Dose response curves plots for EDO 46, 92 and 74 against 4 chemotherapy drugs and Mubritinib. (**B**) Heatmap and hierarchical clustering representing the 1-AUC values of each EDO against a single drug. “C”: Complete response in the patient to induction therapy, “N” No induction therapy in the patient, “P” Partial response in the patient to induction therapy.

We treated each organoid with a single chemotherapy agent, each of which are part of a clinically utilized induction regimen (Fig. 2A). We elected to use a prototypical agent for each class (e.g. cisplatin for platins and paclitaxel for taxanes). Hence, for CROSS, we looked at the response to Cisplatin and Paclitaxel, whereas for FLOT, we assessed the response to 5FU, Cisplatin, and Paclitaxel. An anthracycline-based triplet (epirubicin, cisplatin, 5-FU) is an often used alternative and thus we included epirubicin/irinotecan. Overall, the organoids displayed various sensitivities to the different drugs, which suggests an individualized response to each chemotherapy regimen. Epirubicin and Paclitaxel had the most effect on the organoid viability (mean AUC: 0.53 and 0.47, respectively) and Irinotecan, 5FU, and Cisplatin had moderate effects (mean AUC: 0.83, 0.83 and 0.81 respectively) (Fig. 2B). Similarities were identified between the patient’s response to treatment and the organoid response. Both patient 46 and 92 received CROSS treatment, but patient 92 had a near complete response whereas patient 46 had a partial response (Table 1). Indeed, EDO 92 was more sensitive to Cisplatin than EDO 46 (Fig. 2A). Paclitaxel response appears similar between EDO 46 and 92. When we compared the EDO 70 (partial response to FLOT) with EDO 82 (near complete response to FLOT), we did not see a difference in response against Cisplatin and Paclitaxel, but EDO 70 was more resistant towards 5FU, Epirubicin and Irinotecan than EDO 82 (Supplementary Fig. S3). Patient 74 did not receive induction therapy so we could not compare the organoid response to the tumor but EDO 74 was extremely sensitive to Paclitaxel (Fig. 2A) and resistant to almost all the other drugs, suggesting that a taxol based treatment might have been effective for this patient. Unsupervised clustering demonstrated that complete responders separated from partial responders (Fig. 2B). The overlap between the organoid and the tumor response may yet be regimen dependent, therefore further work will need to be done to validate these preliminary results.

### Personalized treatment approach using organoids

**Figure 3:**
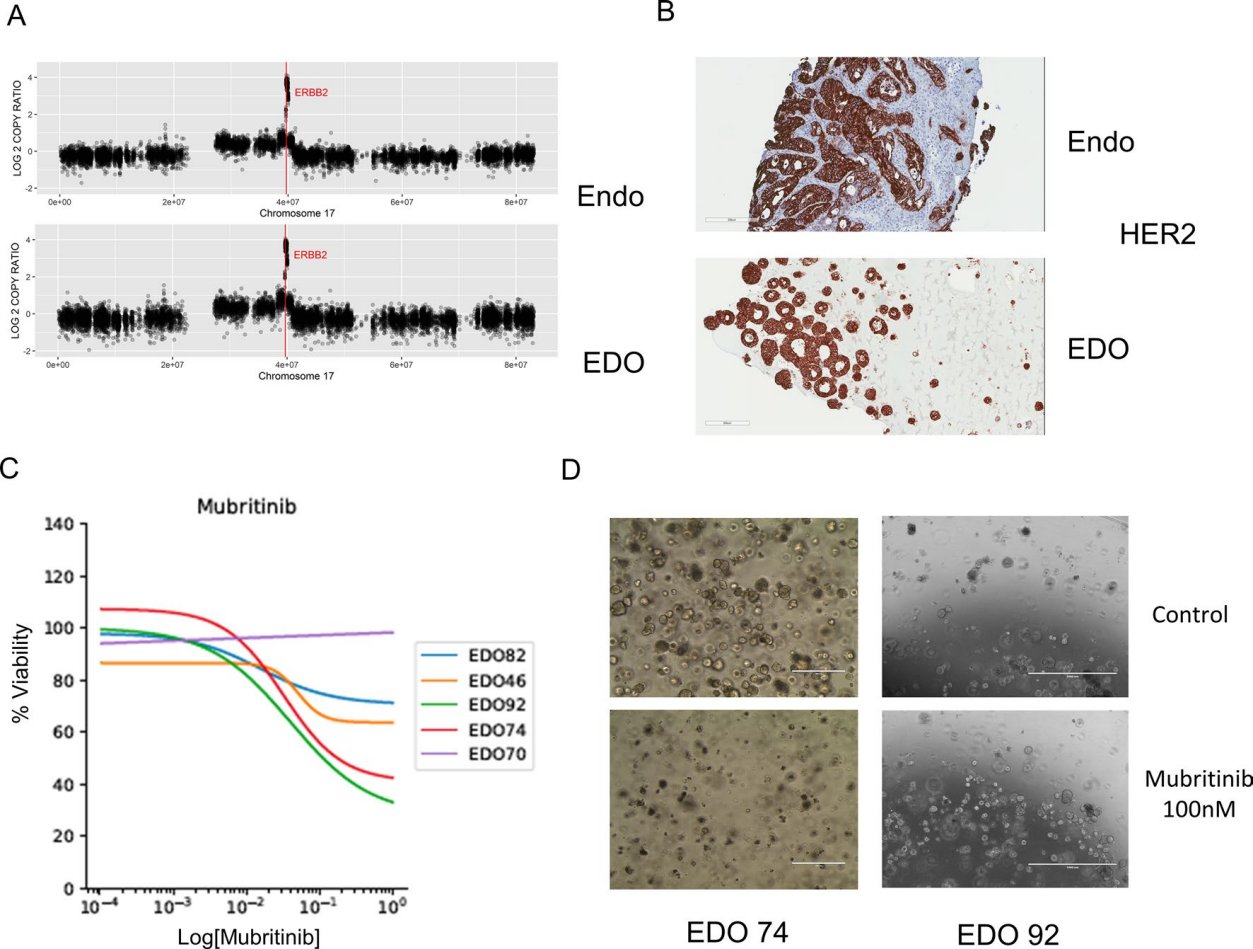
EDO can be used to test patient specific treatments. (**A**) Copy number variation on chromosome 17 for patient 74. ERBB2 amplification is highlighted for both endoscopy and EDO. (**B**) Representative picture of HER2 IHC on endoscopy and EDO. (**C**) Mubritinib dose response for all five EDOs. (**D**) Bright light microscopy pictures of EDO 74 and 92 treated with and without Mubritinib (100 nM) for 7 days.

We next sought to test whether EDO genomics can help guide the use of targeted therapies. For EAC, trastuzumab for HER2 overexpression is the sole targeted therapy in clinical use and HER2 overexpression is rare. Indeed, out of our 5 patients, only one was HER2 positive (patient 74) (Table 1). This patient had an amplification of the ERBB2 region present in both the tumor and EDO (Fig. 3A). To validate this result, we performed HER2 immunohistochemistry and both the tumor and EDO from patient 74 stained positive (Fig. 3B). Since the EDO recapitulated the HER2 expression of the tumor, we explored if the organoid will respond to a anti-HER2 treatment. In order to mimic the transtuzumab effect on the tumor, we tested the effect of a HER2 specific drug (Mubritinib) on all our organoids. As expected, EDO 70, 82, and 46 showed no response and EDO 74 showed a moderate response (Fig. 3C). Furthermore, when cultured over 7 days, we observed cell death in EDO 74 culture (Fig. 3D).

Unexpectedly, EDO 92 organoid responded in a similar manner to EDO 74 despite being HER2 negative. No HER2 amplification was observed in the endoscopy biopsy and EDO from patient 92 and furthermore, they were both HER2 negative when assessed by immunohistochemistry (Supplementary Fig. S3). A recent paper described a secondary effect of Mubritinib as an electron transport chain (ETC) complex I inhibitor^12^. Since our drug assay depends upon ATP, it may be that the effect we observed in EDO 92 is due to the Mubritinib effect on ETC. Indeed, when we cultured EDO92 for 7 days with Mubritinib, we did not observe cell death, in contrast to the EDO74 response (Fig. 3D).

### Barrett’s esophagus cells represent a potential source of contamination for esophageal adenocarcinoma organoids

**Figure 4:**
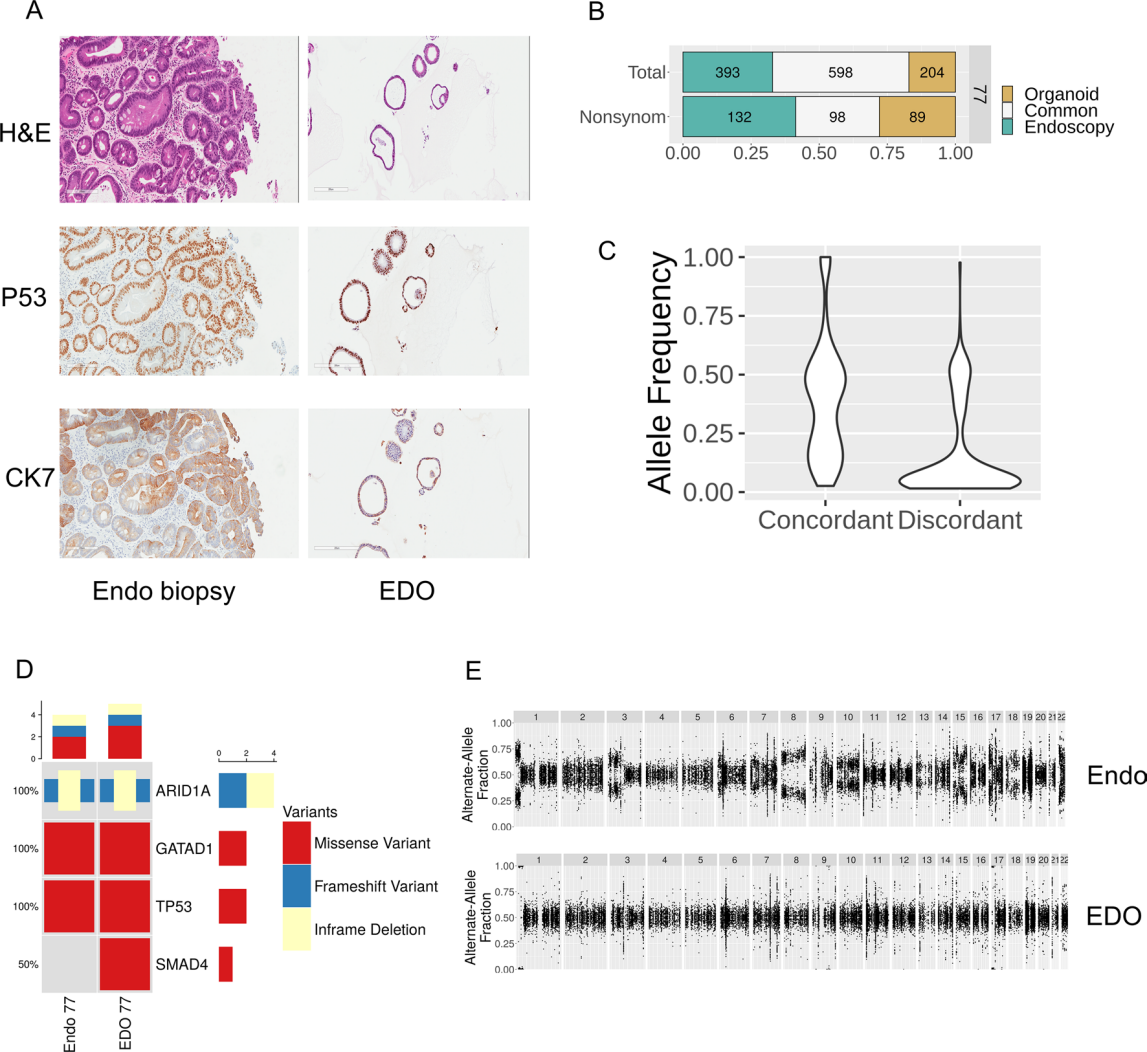
Barrett cells represent a potential contaminant source in EDO. (**A**) Representative images of H&E and IHC of P53 and CK7 of patient 77 endoscopic biopsy and EDO. (**B**) Concordance and discordance plots of SNV for patient 77 endoscopic biopsy and EDO. (**C**) Violin plots of the frequency plots of concordant and discordant SNV for both endoscopy tumors and EDO. (**D**) Oncoprint table for endoscopy and EDO samples from patient 77. (**E**) Copy Number Variation plots of patient 77 paired sample (endoscopy and EDO).

One of the major issues when establishing cancer organoids is contamination from normal tissue^13^. In EAC, this concern is enhanced by the potential presence of Barrett’s cells alongside the normal squamous cells in the esophageal epithelium. Our culture condition is designed to favor the growth of columnar type cells over squamous cells. BE stem cells share features with gastric and intestinal stem cells^14^ and therefore can grow in our organoid medium^15^. Thus, they represent a more threatening source of contamination than normal esophageal cells. More troublesome is also the fact that dysplastic BE is known to harbor mutations similar to bona fide EAC. We believe that we observed this scenario with one of our EDO. EDO 77 was histologically different than the others with a very cystic appearance (Fig. 4A) and slow growth rate after passage 6 (data not shown). In terms of immunohistochemistry, EDO77 was positive for P53 and faintly stained positive for CK7, similar to the endoscopic biopsy (Fig. 4A). EDO 77 had similar SNV concordance to its corresponding tumor (Fig. 4B,C) and similarly had TP53 and MUC6 mutations (Fig. 4D). Histology and immunohistochemistry on the endoscopic biopsy revealed that it was composed almost primarily of high-grade dysplasia (HGD) cells; tumor was present, but in a small amount (Fig. 4A). We therefore suspected that EDO 77 was derived from Barrett’s high grade dysplastic cells. The CNV profile of EDO 77 supported our suspicion about the nature of EDO77 in that the cells were largely diploid and lacking the structural rearrangements typically seen in EAC (Fig. 4E). Despite the lack of conclusive proof, this evidence suggests that EDO77 is a Barrett’s organoid not an EAC organoid.

## Discussion

In this report, we describe our protocol to successfully create EAC organoids derived from naïve endoscopic biopsies. We undertook this project because of the limitations of the current models of EAC and also because of the limited avenues for personalizing care for patients with EAC.

One of the major flaws of pre-clinical EAC models such as PDX has been the poor establishment rate when compared to other disease sites. With our protocol, we were able to achieve a 57.2% culture rate which is higher than EAC PDX and also the organoid protocol described by Li et al.^10^. Li et al. generated patient derived organoids (PDO) from resection specimens that underwent induction therapy and this may have hindered their culture rate; the induction therapy may have been successful at eradicating the tumor. Naïve endoscopic samples may therefore lead to higher establishment rates, but present alternative challenges. Endoscopic biopsies are only 0.5 mm^3^ and while we can take samples extremely fresh; i.e., biopsy forceps directly into the culture media, patients with EAC will often have dysphagia and therefore patient-derived bacteria and yeast contamination becomes a more pressing concern. We have partially mitigated this by using a different media containing a custom antimicrobial cocktail to store the biopsies prior to culturing for organoids. Among the samples which did not grow, poor tumor content may be a major cause of the non-growth. Because of the biopsy size, tumor cellularity is a critical aspect for the establishment of endoscopic organoids.

We next examined the recapitulation of the resultant organoids to the originating tumor. We observed around a 60% SNV overlap, which despite being lower than ones described in gastric or colorectal cancer, is similar to Li et al.^10^. It is possible that the lower mutation overlap between the tumor and organoid in EAC is a reflection of the known heterogeneity of those tumors. Indeed, shared mutations between the organoid and tumor showed a much higher allele frequency in the organoid, suggesting a purer cell population following organoid culture. Moreover, SNV unique to organoids were much lower and therefore could represent de novo mutations or, more likely, low frequency tumor SNVs that were amplified through organoid culture. We attempted to mitigate this computationally by comparing high confidence organoid SNV calls to tumor SNV calls, as heterogeneity of the tumor may have reduced the SNV frequency below the confidence filter.

As previously described in large scale genomic studies of EAC^16, 17^, there was a high mutational burden but few that were recurrent. Indeed, in our population, overlap with the previous gene list described was minimal^11^. There were only 32 genes which were recurrently mutated in both the tumor and organoid in 2 or more pairs and 5 which were recurrently mutated in 3 or more pairs. None shared the same SNV and one of these genes was TTN, a large gene and therefore probabilistically higher rate of mutation. This further supports the hypothesis that EAC is a cancer of structural rearrangement rather than that of specific mutations.

We therefore looked at copy number variation similarities between the originating tumor and resulting organoids. While we observed similarities in ploidy and copy number aberrations between tumors and organoids, we found that we had a very poor tumor purity in 2 of our samples. This is a troubling issue with endoscopic samples as biopsies are taken at the discretion of the endoscopist. Laser capture microdissection might be required for samples with low tumor purity but the small amounts of tissue will likely limit this approach. It may be that frozen sections will need to be taken at the time of endoscopic biopsy to confirm adequate tumor amounts before being used. Additionally, use of whole genome sequencing may facilitate some of this analysis in the future.

As we only have 5 EDOs, we cannot generalize our drug response results or make any meaningful statistical analysis. Moreover, we are comparing the effect of only a single drug to a multiple drug regiment in patients. Despite these discrepancies, we observed variations in the response to drugs between organoids, with epirubicin and paclitaxel having the most effect and 5FU the least. When we compared the corresponding organoid responses to the patient’s regimens, the organoids responded similarly to the patients. Interestingly, the organoids from the partial response patients had a higher overall resistance compared to patients who had a near complete response, suggesting that some tumors may simply be more resistant to current chemotherapeutics. Obviously, this is only the first step and the use of clinically-relevant drug combinations will be needed to model current chemotherapy regimens. However, these preliminary results are encouraging and the systematic culture of EDOs for drug testing is in progress.

Given the lack of recurrent mutations in this disease, one could argue that personalized targeted treatment is most needed in this population. Vlachogiannis et al. have recently shown that colorectal cancer organoids derived from a patient sensitive to the chemotherapeutic drug regorafenib responded better than organoids derived from a patient resistant to the same drug^18^. We therefore tested Mubritinib, a small molecule inhibitor of HER2, on the EDOs. HER2-positive EDOs responded to Mubritinib and HER2-negative EDOs did not. One HER2-negative EDO appeared to respond to Mubritinib based on our assay but brightfield imaging did not show any cell death. This may represent the recently described direct and ubiquinone dependent ETC complex I inhibitor effect of Mubritinib interfering with our ATP-dependent assay.

Finally, Barrett’s esophagus cells have the ability to form organoids, and therefore can be a source of contamination^15^. We describe our experience with an organoid that had mutations but very little CNV and failed to grow beyond passage 6. When we stained the adjacent biopsies used to generate the organoid, there was significant Barrett’s esophagus seen, suggesting that the organoid originated from Barrett’s cells. Indeed, Barrett’s cells are columnar and can carry a significant burden of somatic mutations and can form organoids^15^. This can make it difficult to separate them from EAC organoids either by culture conditions, e.g. use of nutlin-3 to remove p53 wild-type cells, or by morphology. From this experience, it seems that one way to validate the nature of the organoids might be to inspect the copy number variation. Some reports have indicated that high grade dysplasia can contain CNVs, but the line between high-grade dysplasia and bona fide malignancy remains blurry. Interestingly, Li et al. were unable to culture organoids from pre-malignant Barrett’s esophagus^10^. Their protocol differs from ours and Sato et al. in that they do not add Gastrin to the medium^15^. Gastrin has been previously shown to control the proliferation of Barrett’s cells^19, 20^. The presence of Gastrin in our medium may therefore enhance the lifespan of BE cells in our organoid culture. When we cultured EDO 77 without gastrin for multiple passages, we observed a clear diminution in the number of organoids. However, there were significant differences in the copy number variation in chromosomes 7 and 18, suggesting that these organoids may represent a rare but malignant clone brought out by removal of gastrin (Supplementary Fig. S4). Perhaps, EDO77 was cultured from Barrett’s with a higher grade of dysplasia, given its proximity to the malignancy, or perhaps this organoid was truly an adenocarcinoma organoid which had unusual properties.

In summary, we demonstrate, for the first time, the ability to create matching EAC organoids from endoscopic biopsies with a high success rate. Despite the small amount of tissue, the EAC organoids created largely recapitulated the originating tumor on histology, molecular characteristics, and sensitivity to drugs and represent a viable and needed clinical model on which EAC can be further studied. Furthermore, there exists a potential to use these organoids as a platform to improve the personalization of EAC treatment by drug testing of conventional and targeted therapies.

## Methods

### Establishment and maintenance of endoscopic biopsy derived organoids

Endoscopic biopsies at the time of diagnosis were collected and stored in PBS containing Penicillin–Streptomycin, Neomycin, Anti-Anti (Gibco, USA) and Primocin (Invivogen, USA) (PBS PNP). The biopsies were then cut into small pieces using a scalpel and washed 3 times in PBS PNP. The remaining pellet was resuspended in Trypsin 0.5% (Gibco, USA) and transferred to a MACS C-tube (Miltenyi Biotec, USA). The tube was then run on a gentleMACS dissociator (MiltenyI Biotec, USA) and filtered through a 70 µm cell strainer. Fresh Advanced DMEM/ F12 (Gibco, USA) medium was added and cells spun down. The pellet was resuspended in Matrigel GFR (Corning, USA) to a concentration of 30,000 cells per 50 µL of Matrigel. 50 µL of cell suspension in Matrigel was plated per well of a 24-well plate (ThermoScientific, USA) and incubated for 10 min at 37 °C for polymerisation after which either Medium A and B were added to the wells. Medium was changed every 48 h with freshly made medium. After passage 6, the organoids were STR tested. A detailed morphological evaluation and comparison of the primary endoscopic biopsies and matched organoids was performed. Passage 6 was chosen to minimize potential normal cell contamination and potentially represents a purer tumor organoid culture.

Medium A: Advanced DMEM (Gibco, USA), Hepes (10 mM) (Gibco, USA), GlutaMax (2 mM) (Gibco, USA), Penicillin–Streptomycin (1x) , Neomycin (1x) , Primocin (1x), Anti-Anti (1x), N-Acetyl l-Cysteine (1.25 mM) (Sigma, USA), B27 (1x) (Gibco, USA), Gastrin (10 nM) (Sigma, USA), EGF (50 ng/mL), Noggin (100 ng/mL) (Peprotech, USA), A-83-01 (0.5 µM) (R&D Systems, USA), CHIR (2.5 µM) (R&D Systems, USA), Rspondin1 conditioned medium(10% v/v), Wnt-3A conditioned medium (40% v/v). The conditioned media were obtained from Princess Margaret Living Biobank.

Medium B: Medium A + SB202190 (10 µM) Selleckchem (Houston, TX).

### Histology and immunohistochemistry

EDO grown in Matrigel were fixed in paraformaldehyde for 2 h and embedded in Histogel (Thermo Fisher Scientific, Waltham, MA). Adjacent fresh tumour tissues and Histogel embedded organoids were then fixed with 10% formalin for 24–48 h followed by fixation in 70% ethanol with containing eosin. Formalin-fixed paraffin embedded tumor tissues and organoids were cut into 4 μm thick slices and allowed to dry overnight at 60 °C. Prepared tissue sections were stained with appropriate antibodies using the BenchMark XT autostainer (Ventana Medical System, Tucson, AZ). Primary antibodies specific to p53 (Leica NCL-p53-D07, clone D07), CK7 (Dako M7018, clone OV-TL12130) and HER2 (Thermo Scientific RM9103, Rabbit monoclonal SP3) were used for IHC analysis. The slides were scanned and imaged using an Aperio Scanscope XT (Leica, Canada).

### Doubling time of endoscopic biopsy derived organoids

EDO were dissociated to single cells, counted, and seeded on a thin layer of Matrigel in 384 well plates (3,000 cells per well) in triplicate. At 24, 48, 72, and 96 h post-plating, relative cell growth was assessed by ATP quantification using the CellTiter-Glo 3D luminescence-based assay (Promega, USA). Growth curves were generated and doubling rate determined.

### Whole exome sequencing and analysis

WES was performed to a target depth of 40×. Library generation was performed with Sureselect Exon V7. Initial quality checks were performed with FastQC v0.11.7^21^ before aligning to the GRCH38 human reference genome using BWA 0.7.17^22^ MEM. Samtools 1.9^23^ flagstat assayed alignment quality. PCR and optical duplicates were marked with Picard MarkDuplicates 2.18.20^24^ and base quality recalibration was performed using GATK 4.1.2.0^25^.

Germline short indels and SNP’s were called and filtered using GATK HaplotypeCaller and VariantFiltration. Somatic short indels and SNV’s were called and filtered using GATK FilterMutect2, Strelka-2.9.2^26^, bcftools 1.9 mpileup, GATK FilterMutectCalls, and GATK VariantFiltration. Somatic variants were further filtered against all germline variants from paired normal. The filtered variants from the tumor were compared to the unfiltered variants from the EDO, allowing for rescue of concordant calls in the organoid that failed to pass filter in the tumor due to a lower read count and confidence in the alternative allele owing to heterogeneity. The reverse was then performed to rescue low confidence tumor variants.

Variants were annotated with ensemble-vep 96.0^27^. EAC relevant calls were identified by comparing organoid and endoscopy nonsynonymous mutations against a list of 76 genes produced from a previous study of 551 EAC cases^11^. Copy ratio alterations and allelic fraction plots were performed using GATK^28^. The ggplot2 package for R was used to create the plots^29, 30^.

### Drug screening assay

For in vitro drug testing, compounds were purchased from Selleckchem (Houston, TX) and dissolved in DMSO. Organoids were dissociated to single cells, counted, and seeded onto a thin layer of Matrigel in 384 well plates (3,000 cells per well) in triplicate for 3 days prior to drug treatment. Organoids were treated with a range of drug concentrations (0.01–10 μM) for 96 h and cell viability was assessed using the CellTiter-Glo 3D assay (Promega, USA). Relative IC50 values were graphed and calculated from 12-point drug concentrations with four-parameter nonlinear logistic equation using using src^31^ and Matplotlib^32^ packages for R and python, respectively.

Normalized AUC values were obtained by dividing the AUC value by the maximum area for the concentration range measured for each drug. The heatmap was generated using using src^31^ and Matplotlib^32^ packages for R and python, respectively.

### Ethics approval and sample collection

The study was approved by the University Health Network Ethics Board (REB#36616 and CAPCR#14-8514.5). Endoscopic and blood samples were collected from EAC patients who consented to tissue collection. Clinico-pathological data are summarized in Table 1. Written informed consent was obtained from all participants at enrolment. Blood samples were collected at time of the endoscopy, centrifuged at 2,000*g* for 5 min, and the buffy coat collected and stored at − 80 °C prior to DNA extraction. The buffy coat was used as the germline control. Similarly, 2 pieces of the endoscopic biopsy were snap frozen and stored at − 80 °C. The DNA extraction was performed using the Qiagen DNeasy Blood and Tissue Kit (Qiagen Inc, Venlo, NL). All methods were performed in accordance with relevant guidelines and regulations.

## Supplementary information

Supplementary Figure Legends.

Supplementary Information 1.

## Abbreviations

EAC: Esophageal adenocarcinoma
EDO: Endoscopy derived organoid
5FU: Fluorouracil
