# Supplementary material for: Towards personalized induction therapy for esophageal adenocarcinoma: organoids derived from endoscopic biopsy recapitulate the pre-treatment tumor
Source: Sci Rep. 2020 Sep 3;10:14514. doi: 10.1038/s41598-020-71589-4 (PMC7471705; doi:10.1038/s41598-020-71589-4)
Supplement: Supplementary file 2 — Supplementary Information 1. [file 41598_2020_71589_MOESM2_ESM.docx]

**Supplementary Figure Legends**

Supplementary Figure 1: (A) H&E of the EDO and matching organoid. (B) Summary table of the TP53 mutations found in EDOs and endoscopic biopsies.

Supplementary Figure 2: (A) Oncoprint table for all 5 paired samples. The gene list was established by selecting for SNV occurring in three or more samples. (Endoscopy and EDO). (B) Copy Number Variation plots of patient 70, 82 and 92.

Supplementary Figure 3: (A) Dose response curves plots for EDO 70 and 82 against 5 chemotherapy drugs and Mubritinib (B) Copy number variation on chromosome 17 for patient 92. ERBB2 amplification is highlighted for both endoscopy and EDO. (C) Representative images of HER2 IHC for endoscopy biopsy and EDO from patient 92.

Supplementary Figure 4: (A) Concordance and discordance plots of SNV for EDO 77 and EDO 77 minus gastrin. (B) Oncoprint table for EDO 77 and EDO 77 minus gastrin samples from patient 77. (C) Copy Number Variation plots of EDO77 minus gastrin.
